# 

**Published:** 1814-03

**Authors:** 


### Medical and Physical Journal.

3 OF VOL. XXXI.] MARCH, 1814. [NO. 181.

### The PROPRIETORS to the PUBLIC.

*IT has been the fate of the Proprietors of this original Journal, in common with the projectors of every successful undertaking, to witness the rise, decline, and fall of many rival works on a similar plan. They do not complain either of circumstances which are not peculiar to themselves, nor of the honorable and well-meant endeavours of others; always confiding for protection, against piratical copyists or insidious oppositions, in the just feelings of that intelligent part of the community to whom this Journal is addressed. They consider it due, however, not less to the* FACULTY *at large than to many old and valued Friends, and their own interests in particular, to assert the superior and undiminished pretensions of a work of established reputation and universal circulation, as a means of drawing together original Communications from all parts of the world, and of giving them that degree of currency which affords at once a maximum of gratification to the writers, and of utility to the profession and the public. On this point they venture also to urge the evident* CONVENIENCY *and* ECONOMY *of uniting all current medical improvements and correspondence in one periodical work; and they presume to urge the legitimacy of their own claims, founded on their* LONG ESTABLISHMENT, *their* PAST SERVICES, *their well-known* IMPARTIALITY *in whatever regards the interests and feelings of various branches of the profession, and their determination to concentrate within their pages* EVERY ORIGINAL FACT *and* VALUABLE OBSERVATION, *let them appear in whatever form or quarter they may.*

*They are duly sensible at the same time that the foundation of all their pretensions to public favor and preference is in the undeviating merit of their work; and they have, therefore, among other new arrangements, associated with* DR. FOTHERGILL *a colleague selected from another branch of the profession, and whose title to the approbation of their readers will, they trust, be made evident by the increased interest of their pages.*

### MONTHLY CATALOGUE OF MEDICAL BOOKS.

MEDICAL Reports on the Effects of Water, Cold and Warm, as a Remedy in Fever and Febrile Diseases. By James Currie, M.D. F.R.S. 2 vols. 8vo. 18s. New edition.

Abernethy on Local Diseases. New edition. 8vo. 7s.

Observations on the distinguishing Symptoms of three different Species of Pulmonary Consumption. By Andrew Duncan, senior, M.D. 8vo. 6s.

Nosology, or a Systematic Arrangement of Diseases in Classes, Orders, Genera, and Species, with accurate Definitions. Translated from the Latin of William Cullen, M.D. 12mo. 2s. 6d.

Observations on the Nature and Treatment of Consumption. By Charles Pears, M.D. F.L.S. 8vo. 4s.

An Essay on Medical Economy. 8vo. 8s.

Lectures on Comparative Anatomy. By Sir Everard Home, Bart. F.R.S. 2 vols. 4to.

On Hydrophobia, its Prevention and Cure, with a Dissertation on Canine Madness. By Benjamin Moseley, M.D. 4to.

---

### *NOTICES TO CORRESPONDENTS.*

*We have been informed that a contagious disease of the eyes, resembling the Egyptian ophthalmia, has appeared in certain districts, and spread through whole families. As such an occurrence must necessarily excite alarm, we are anxious to afford the most early intelligence upon the subject. We therefore intreat the attention of our correspondents to this important object, and request they will favor us with their remarks; and at the same time authenticate their communications with their names.*

*Mr. Rigby's paper on Vaccination arrived too late for insertion this month, but will appear in the next Number of the Journal, with several other valuable communications which have already come to hand.*

